# The effect of diabetes self-management education on HbA1c and quality of life in African-Americans: a systematic review and meta-analysis

**DOI:** 10.1186/s12913-018-3186-7

**Published:** 2018-05-16

**Authors:** Amy T. Cunningham, Denine R. Crittendon, Neva White, Geoffrey D. Mills, Victor Diaz, Marianna D. LaNoue

**Affiliations:** 1 Department of Family and Community Medicine, Sidney Kimmel Medical College, Thomas Jefferson University, 1015 Walnut Street, Suite 401, Philadelphia, PA 19107 USA; 2 Jefferson College of Population Health, Thomas Jefferson University, Philadelphia, PA USA; 3 Center for Urban Health, Thomas Jefferson University Hospital, Philadelphia, PA USA

**Keywords:** Type 2 diabetes, Diabetes self-management education, African-Americans, Disparities

## Abstract

**Background:**

Type 2 diabetes presents a major morbidity and mortality burden in the United States. Diabetes self-management education (DSME) is an intervention associated with improved hemoglobin A1c(HbA1c) and quality of life(QOL), and is recommended for all individuals with type 2 diabetes. African-Americans have disproportionate type 2 diabetes morbidity and mortality, yet no prior meta-analyses have examined DSME outcomes exclusively in this population. This systematic review and meta-analysis examined the impact of DSME on HbA1c and QOL in African-Americans compared to usual care.

**Methods:**

Randomized controlled trials, cluster-randomized trials, and quasi-experimental interventions were included. 352 citations were retrieved; 279 abstracts were reviewed, and 44 full-text articles were reviewed. Fourteen studies were eligible for systematic review and 8 for HbA1c meta-analysis; QOL measures were too heterogeneous to pool. Heterogeneity of HbA1c findings was assessed with Cochran’s Q and *I*^2^.

**Results:**

HbA1c weighted mean difference between intervention and usual care participants was not significant: − 0.08%[− 0.40–0.23];*χ*^2^ = 84.79 (*p* < .001), *I*^2^ = 92%, (*n* = 1630). Four of five studies measuring QOL reported significant improvements for intervention participants.

**Conclusions:**

Meta-analysis results showed non-significant effect of DSME on HbA1c in African-Americans. QOL did show improvement and is an important DSME outcome to measure in future trials. Further research is needed to understand effectiveness of DSME on HbA1c in this population.

**Trial registration:**

PROSPERO registration: CRD42017057282.

**Electronic supplementary material:**

The online version of this article (10.1186/s12913-018-3186-7) contains supplementary material, which is available to authorized users.

## Background

Type 2 diabetes is responsible for a staggering morbidity and mortality burden. As of 2015, 9.4% percent of the United States population has diabetes; 95% of these individuals have type 2 diabetes [1]. Type 2 diabetes is associated with microvascular complications such as retinopathy, neuropathy and nephropathy, and with higher risk of macrovascular complications, including coronary artery disease, peripheral arterial disease, and stroke. Currently, type 2 diabetes is the seventh-leading cause of death in the United States [1].

Furthermore, profound racial and ethnic disparities exist in type 2 diabetes morbidity and mortality in the United States, particularly for African-Americans. Currently, 12.7% of African-Americans have type 2 diabetes [1]. African-Americans are less likely to have controlled HbA1c than non-Hispanic whites [2], are also more likely to develop retinopathy and nephropathy [3], and more likely to be hospitalized with diabetes-related complications [4]. African-Americans with type 2 diabetes also report higher levels of diabetes-related distress than non-Hispanic whites [5]. Ultimately, African-Americans have the highest diabetes-related mortality rates of any racial or ethnic group in the United States [3].

These Type 2 diabetes disparities result from a complex mix of factors. Low birth-weight and maternal-fetal stress are more common in African-American children and increase the risk of developing type 2 diabetes [6]. Higher type 2 diabetes prevalence and poorer HbA1c control may result from ethnic differences in obesity rates, body fat distribution, and glucose metabolism [6]. Cultural food practices and customs may also pose a challenge to diabetes management, such as consumption of breaded and fried meats and simple carbohydrates [7]. Additionally, African-Americans are disproportionately affected by socioeconomic factors such as poverty, poorer quality housing, lack of neighborhood spaces for physical activity, and limited access to healthy food [8]. Health care access barriers and lower quality of care also contribute to poorer diabetes outcomes in African-Americans, as can patient-provider racial discordance, perceived racial bias in medical encounters, and resulting patient mistrust in healthcare providers and systems [9].

Self-management of type 2 diabetes requires regular blood glucose monitoring, management of diet, physical activity, medications, and ongoing medical care. A key goal of diabetes self-management is the control of hemoglobin A1c (HbA1c), which is a measure of average blood glucose over several months. Poorly-controlled HbA1c is associated with microvascular and macrovascular complications [1]. The demands of managing this complex illness also affect many dimensions of quality of life (QOL), which encompasses physical, emotional and social well-being. Individuals with diabetes report lower QOL than individuals without chronic illnesses [10]. Contributors to lower QOL include diabetes-related distress; in the recent Diabetes Attitudes, Wishes and Needs second (DAWN2) study, 44.6% of those with type 2 diabetes reported distress regarding hypoglycemic events, physical health, emotional well-being, and financial strain [11]. In turn, lower QOL affects the ability to manage HbA1c and other diabetes care activities [12].

Recognizing the many challenges of managing type 2 diabetes, the American Diabetes Association (ADA) recommends that all individuals receive diabetes self-management education (DSME) at the time of a type 2 diabetes diagnosis, as well as ongoing self-management support as needed [13]. The goal of DSME is to increase an individual’s self-efficacy to manage diet, physical activity, glucose monitoring, stress management, and other necessary skills and behaviors for successful diabetes outcomes [13]. Meta-analyses have established the impact of DSME on glycemic control and QOL. In a 2002 meta-analysis, DSME participants demonstrated reductions of 0.76% in hemoglobin A1c (HbA1c) at immediate follow-up, with reductions in HbA1c attenuating to 0.24% at follow-up points 4 or more months post-intervention. The authors found three interventions measuring QOL, two of which showed QOL improvements in DSME participants; they did not combine these studies in a meta-analysis [14]. A more recent meta-analysis of group DSME programs showed HbA1c declines of 0.44% six months post-intervention, and 0.46% at 12 months. Three studies were eligible for a QOL meta-analysis; QOL changes were not significant, but the authors stipulated that the heterogeneity of the included studies was high [15]. However, neither meta-analysis examined outcomes by racial/ethnic group.

Increasingly, attention has been paid to the differential impact of DSME in racial and ethnic minority groups—including African-Americans--and development of DSME that is culturally-adapted for the language, beliefs, values, and customs of particular groups. In their DSME position statement the (ADA), the American Association of Diabetes Educators (AADE) and Academy of Nutrition and Dietetics call for DSME that addresses a patient’s “cultural needs,” [13] and the AADE lists provision of “culturally competent supportive care across the lifespan” as a competency for diabetes educators [16]. Nam et al.’s 2012 meta-analysis of 12 culturally-tailored DSME interventions—four of which targeted African-Americans—showed an effect size of − 0.29 on HbA1c [17], indicating a small effect. A 2014 meta-analysis of the impact of DSME on HbA1c in ethnic minorities found an overall 0.31% HbA1c reduction in the 39 included studies; 33% of these studies included African-Americans [18]. However, these meta-analyses did not explore HbA1c results for African-Americans separately, nor did they examine QOL as an outcome.

Despite the higher type 2 diabetes morbidity and mortality burden in African-Americans, no systematic reviews or meta-analyses have specifically analyzed the impact of DSME on two critical measures--HbA1c and QOL-in this population. Further, none have examined whether certain DSME characteristics, such as number of contact hours or culturally-adapted interventions, might result in better outcomes for African-Americans. The purpose of this systematic review and meta-analysis is to examine the impact of DSME in African-American adults with type 2 diabetes mellitus on HbA1c and QOL. Subgroup analyses also examined the impact of several DSME characteristics, including cultural adaptations, on HbA1c.

## Methods

The systematic review and meta-analysis study protocol was developed prospectively and reported using Preferred Reporting for Systematic Review and Meta Analyses (PRISMA) guidelines [19]. The systematic review and meta-analysis procedures used were developed in consultation with the Cochrane Handbook for Systematic Reviews of Interventions [20]. The protocol was registered at the international prospective register of systematic reviews (PROSPERO) (ID: CRD42017057282) [21].

### Search strategy

The search strategy, including databases used and search terms, was developed in consultation with a medical librarian. An initial search was developed for OVID MEDLINE using keywords, medical subject (MeSH) terms and publication types based on the PICO framework (participants, comparison, intervention, and outcomes). Participants were African Americans (“African Americans,” “African Americans”[MeSH] with type 2 diabetes (“type 2 diabetes,” “type 2 diabetes mellitus,” “diabetes,” “T2DM”(type 2 diabetes mellitus), “Diabetes Mellitus”[MeSH], “Diabetes Mellitus, Type 2”[MeSH], “NIDDM” (Non-insulin dependent diabetes mellitus), or “Non-insulin dependent diabetes mellitus.”) The intervention was DSME (“diabetes self-management education,” “self management education,” “DSME,” “education.” “health education,” “diabetes education,” “Patient Education as Topic”[MeSH], or “Self Care”[MeSH]); the comparator was a control group in a randomized-controlled trial or quasi-experimental study with matched controls (“randomized controlled trial.” “controlled clinical trial,” “randomly,” “randomized,” “trial,” “control,” “groups,” or “quasi-experimental”). Outcomes were HbA1c (“HbA1c,” “A1c,” “glycemic control,” “Hemoglobin A, Glycosylated”[MeSH], or “hemoglobin A1c protein, human”[MeSH]) and QOL (“HRQL,” “QoL,” “health-related quality of life,” “Quality of Life”[MeSH]), “QOL tools OR questionnaires OR surveys,” “SF-36,” “WHOQOL,” “DQOL.” “well-being,” “psychological well-being,” or “emotional well-being”). A sample OVID MEDLINE search strategy may be found in Additional file 1.

Databases searched were OVID MEDLINE, Ovid Eric, PsycINFO, Scopus, CINAHL EBSCO, and the Cochrane Central Register of Controlled Trials. To minimize the potential omission of relevant studies, the citation lists of included studies were reviewed to identify additional studies for potential inclusion. Additionally the tables of contents for selected journals (*Diabetes Care*, *The Diabetes Educator*, *Annals of Internal Medicin*e, and *Annals of Family Medicine*) were hand-checked. The search strategy also included grey literature sources such as non peer-reviewed government and nonprofit publications (the Agency for Healthcare Research and Quality, the ADA, and the Centers for Disease Control and Prevention).

### Inclusion and exclusion criteria

All citations were reviewed against pre-determined inclusion and exclusion criteria for eligibility in the systematic review. Included study designs were randomized-controlled trials or quasi-experimental studies with a matched control group comparing DSME to usual care. The inclusion of quasi-experimental study designs was consistent with the Cochrane Consumers and Communication Review Group standards for evaluation of complex interventions [22]. “Usual care” could consist of usual primary care, assignment to a wait-list, or a minimal educational intervention. The definition of DSME was based on the ADA and AADEs’ National Standards for Diabetes Self-Management Education and Support; e.g., a program to “facilitate the development of knowledge, skills, and abilities that are required for successful self-management of diabetes” [13]. Further, the intervention needed to support at least one of the AADE7 Self-Care Behaviors: healthy eating, being active, monitoring, taking medications, problem solving, healthy coping, and reducing risks [23].

Participants were African-American adults with type 2 diabetes mellitus; to be included, interventions either needed to have exclusively African-American participants, or to report the outcomes for African-American participants separately. All potential settings (clinics, hospitals, community settings, virtual/telehealth/phone, or combinations) were included. Studies selected for the systematic review were eligible for inclusion in the HbA1c meta-analysis if they measured HbA1c mean and standard deviation both pre- and post-intervention; similarly, studies included in the systematic review were eligible for inclusion in the QOL meta-analysis if they measured QOL mean and standard deviation both pre- and post-intervention.

Studies were excluded if: 1) the study population was not exclusively African-American or results for African-Americans are not reported separately; 2) the study had participants with type 1 diabetes, unless type 1 and type 2 diabetes results are reported separately; 3) the study control groups received anything other than usual care; 4) the intervention targeted providers or systems, rather than patients; 5) the intervention was a diabetes disease management or care management intervention, rather than DSME (for example, studies focusing exclusively on medical nutrition therapy or disease management); or 6) the study did not measure either HbA1c or QOL as an outcome. A study was defined as measuring QOL if it used one or more general or diabetes-specific QOL measures, which were pre-specified through a comprehensive literature search using keywords and phrases related to quality of life and frequently-used synonyms (diabetes and “quality of life” or “health-related quality of life” or “psychosocial adjustment” or “distress”). There were no study exclusions based on participant age or sex, article language, or publication date.

### Study selection

Two independent reviewers (AC and DC) conducted the selection process through each phase of the review. All citations identified through the search were imported into a shared bibliography, and duplicate records of the same report were removed. The reviewers independently extracted information from the abstracts into structured evidence tables based on the pre-determined inclusion and exclusion criteria. Based on these criteria, they independently assessed the abstracts’ eligibility for full-text review. The two reviewers compared their results and reached consensus; a third reviewer (ML) served as a tiebreaker when needed. From this process, articles were selected for full-text review. The two reviewers independently read and assessed the full-text articles using the inclusion and exclusion criteria and met to compare results and reach consensus, with the third reviewer serving as a tiebreaker. Through this full-text review, the reviewers identified the final set of articles eligible for inclusion in the systematic review.

### Data extraction

For the articles included in the systemic review, the two reviewers extracted further study data for inclusion in a structured evidence table. Descriptive categories included source citation, number of participants, mean participant age, percentage of participants who were African-American, and study design. Reviewers also recorded whether the intervention was group or individual-based, intervention content, presence of cultural tailoring (according to the studies’ authors), the intervention’s definition of usual care, duration, number of contact hours, provider type, DSME topics addressed, and attrition rate. The HbA1c and QOL measures used, HbA1c/QOL measurement frequency, and results were also recorded.

### Bias and quality appraisal

Risk of bias was examined as an outcome across studies using the Cochrane Collaboration’s Risk of Bias tool, which assesses the presence of biases that pose threats to internal validity [24]. Types of bias examined in the Cochrane Risk of Bias tool included selection bias (random sequence generation and allocation concealment), performance bias (blinding participants and researchers to the intervention a participant receives), detection bias (blinding of outcome assessment from knowledge of what intervention a participant received), attrition bias, reporting bias, and other bias [24]. Studies were judged to have a low, high, or unclear risk of bias for each of these criteria. Quasi-experimental studies were automatically designated to have a high risk of bias on the random sequence generation item of the tool [22]. Two reviewers (AC and DC) independently assessed study bias and then met to compare results and reach consensus. Although assessment of publication bias was included in our protocol, due to the small number of studies in our HbA1c meta-analysis, publication bias could not be assessed. When fewer than ten studies are included in a meta-analysis, tests for forest plot asymmetry are not recommended due the low power to detect a real asymmetry [25].

The overall quality of included studies was assessed using the Grading of Recommendations, Assessment, Development, and Evaluation (GRADE) criteria. In the GRADE system, evidence can be rated as high, moderate, low, or very low. Randomized controlled trials begin with a rating of high quality, and observational studies with a grade of low quality. Factors that can lower a quality rating include limitations in design and execution, heterogeneity (inconsistency of results), indirectness (research does not measure desired intervention or outcomes), imprecision (few patients or events), and publication bias. Factors that can increase a rating include a large magnitude of effect, a dose-response continuum, and plausible residual confounding in observational studies [26]. Two reviewers assessed study quality independently (AC and DC) and met to reach consensus.

### Meta-analysis

All analyses were performed in Review Manager version 5.2 [27]. For studies containing both pre-and post-intervention HbA1c levels, these values were extracted as mean ± standard deviation. First, a meta-analysis was conducted to assess possible baseline HbA1c differences between intervention and control groups. Next, the mean HbA1c for both intervention and control groups at the conclusion of the intervention was transformed into a weighted mean difference (WMD), in which the contribution of each study to the mean difference is weighted by its sample size, and 95% confidence intervals (CIs) were calculated and combined in a random-effects meta-analysis. A random-effects meta-analysis is appropriate when combining studies with differences in the treatment effect [20]. A forest plot was also generated for the HbA1c WMD.

Study heterogeneity was explored using Cochran’s Q and *I* ^2^, with *p* < .05 for Cochran’s Q and *I* ^2^ ≥ 50% indicating substantial heterogeneity [28]. In addition, several subgroup analyses were conducted for HbA1c. First examined was the impact of culturally-adapted versus non culturally-adapted DSME based on the authors’ descriptions of their interventions. Additionally, subgroup analyses were conducted based on intervention contact hours (< 10 versus ≥10), given that 10 or more contact hours has been shown to lead to better DSME outcomes; DSME provider type(s) (e.g., individual (physician, nurse, dietician, pharmacist, health educator), or multiple provider types), individual, group, or combination individual/group DSME, and attrition rate. For QOL, studies with pre-and post-intervention QOL mean ± standard deviation were eligible for inclusion in a meta-analysis.

## Results

**Fig. 1:**
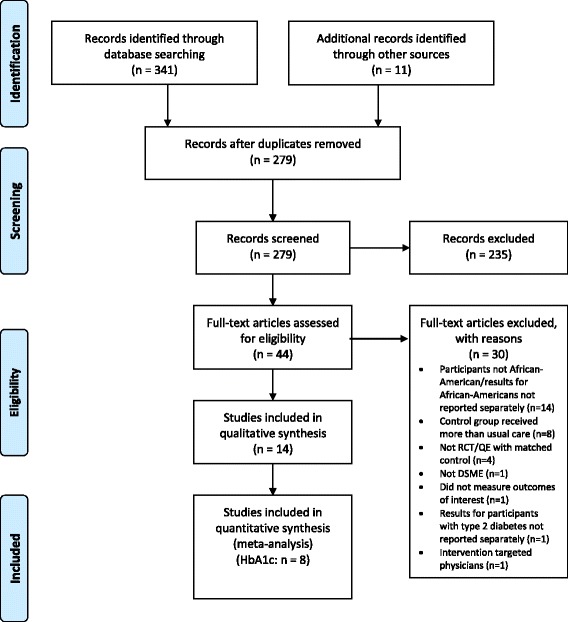
PRISMA Flow Diagram

Fig. 1 shows the PRISMA diagram for the study selection process. A total of 352 citations were retrieved from OVID MEDLINE, Ovid Eric, PsycINFO, Scopus, CINAHL EBSCO, Cochrane Central Register of Controlled Trials, grey literature, and hand searches. After removing duplicates, 279 abstracts remained. After abstract review, 44 articles were selected for full-text review. Ultimately, 14 of those 44 articles were eligible for inclusion in the systematic review; all were from the peer-reviewed literature [29–42].

Table 1 displays the characteristics of interventions included in the systematic review. Publication dates ranged from 1997 to 2015. Ten were randomized-controlled trials, [29–31, 33–36, 38, 40, 42] two were cluster-randomized trials, [32, 39] and two were quasi-experimental studies [37, 41]. Thirteen of the studies exclusively enrolled adult African-Americans with type 2 diabetes; one study recruited both African-American and Hispanic adults with type 2 diabetes, but reported findings on the two racial/ethnic groups separately [38]. The mean participant age was 59. In all studies, more than half of the participants were female; two studies only included female participants [36, 42].

**Table 1:** Included Study Characteristics

Citation	Sample (N)	African-American (%)	Female (%)	Mean Age	Design	Intervention Characteristics: (Duration, Contact Hours, Group vs. Individual, Provider Type)	Control	Attrition Rate	Cultural Tailoring?	HbA1c, QOL Measures
Agurs-Collins et al., 1997 [29]	66	100	77	62	RCT	12 weekly group sessions (60 mins nutrition, 30 mins exercise) and 1 individual diet counseling session over first 3 months; 6 bi-weekly sessions over next 3 months. Providers: Registered dietician and exercise physiologist	1 class within 3 weeks of enrollment (glycemic control); 2 nutritional mailings at 3 and 6 months	15%	Yes	HbA1c: 3×’s: screening/randomization, 3 month visit, 6 month visit; QOL: N/A
Amoako et al., 2008 [42]	68	100	100	61	RCT	4 weeks of phone interventions (1 x per week, from 10 to 60 min) that included 4 phases: Warm-up, assessment of problems, uncertainty appraisal, discussion of strategies to manage uncertainty. Provider: nurse practitioner	Usual care -regular primary care and specialist visits; support group meetings; diabetes management classes	7.35%	No	HbA1c: No specific measurements mentioned, QOL: 2×’s: baseline, 6 weeks post baseline; (Tools: Michel Uncertainty in Illness Scale; Problem Areas in Diabetes Survey).
Anderson et al., 2005 [30]	239	100	82	61	RCT	6 weekly 2 h group sessions; then option for monthly support group, or receive a monthly phone call. Provider: diabetes educator	Wait-listed usual care, no description given.	6.41%	Yes	HbA1c: 4×’s:screening, after 6 week intervention and 6 week control period, 6 months post treatment, 1 year post treatment;QOL: 2×’s: baseline, 6 weeks; (Tool: Diabetes Empowerment Scale Short-Form (DESSF)).
Anderson-Loftin et al., 2005 [31]	97	100	76.5	49	RCT	4 weekly classes in low fat dietary strategies; 5 monthly peer-group discussions, and weekly phone follow up. Provider: diabetes educator	Referral to a local 8 h traditional diabetes class	34.02%	Yes	HbA1c: 2×’s: baseline, 6 months post treatment; QOL: N/A
Bray et al., 2013 [32]	727	100	64.5	60	RCT	Each patient seen 4 times over a 12 month period by the nurse, pharmacist, or dietician care manager for 30 to 60 min; follow-up with case manager every 3 to 6 mos for 2 ys. Providers: nurse, pharmacist and diabetes educator	One 15 min office visit to a physician, nurse practitioner, or physician assistant for labs; frequent diabetes educational handouts received.	7.02%	Yes	HbA1c: 3×’s: baseline, 18 months, 36 months, QOL: N/A
Carter et al., 2011 [33]	47	100	63.8	51	RCT	Telehealth nurse visits: bi-weekly, 30 min video conferencing; DSME modules with social networking to share coping strategies, ask questions. Provider: nurse educator	Usual care from providers	63%	No	HbA1c: 2×’s: baseline, conclusion of 9 month study QOL: 2×’s: baseline, conclusion of 9 month study;(Tool: not specified)
Gaillard et al., 2015 [34]	96	100	70	60	RCT	Didactic lectures at 1–2 week intervals for 6 months; individual one-on-one counseling; trained community health worker support via weekly phone calls and community resources; quarterly point of care physiological testing. Providers: Diabetes educators, registered dietitian, diabetologists,and community health worker	Usual care -anthropometric and metabolic measurements at quarterly intervals	21%	Yes	HbA1c: 3×’s: baseline, 3 months, 6 months QOL: 2×’s: baseline, 6 months; (Tools: Questionnaire diabetic quality of life, 12-Item Short Form Survey (SF-12), diabetes attitude).
Gary et al., 2004 [35]	186	100	76	59	RCT	4 arms: A) usual care; B) usual care + nurse case manager (45 min face-to-face or phone); C) usual care + community health worker (45–60 min face-to-face or phone); D) usual care + nurse case manager + community health worker (3 visits with each educator per year). Providers: nurse care manager, community health worker	Ongoing care from patient’s own health care provider, quarterly newsletter	16%	Yes	HbA1c: 2×’s: baseline; 2 yr. follow up QOL: N/A
Keyserling et al., 2002 [36]	200	100	100	59	RCT	3 arms: (Group A) clinic and community-based center; (Group B) clinic only; (Group C) minimal intervention. Groups A and B received 4 monthly visits with a nutritionist at clinic. In addition, Group A received 3 group sessions at community based center and 12 monthly peer phone calls.. Providers: physical activity leader, peer counselor	Received mailed pamphlets	15%	Yes	HbA1c: 3×’s: baseline, 6 months, 12 months QOL: 3×’s: baseline, 6 months, 12 months; (Tools: Mental Well-Being, Social Well-Being).
Peña-Purcell et al., 2015 [37]	103	100	79.5	63	QE	6 week group educational sessions. Providers: Trained registered nurse, registered dietitian, or a certified diabetes educator	Original study design: Wait-listed control group; due to lack of participants in control group, pre/post design utilized with intervention group.	44%	Yes	HbA1c: 2×’s: baseline, 12 weeks (3 mos); QOL: 2×’s: baseline, 5 weeks; (Tools: Psychological Distress Scale; Healthy Days Measure Scale).
Ruggiero et al., 2014 [38]	266	52.6	68.8	53	RCT	12 months medical assistant coaching for DSME; quarterly in-person contact at regular clinic visits, monthly follow-up phone calls. Providers: medical assistants	Treatment as usual: regular visits with primary care, referrals for specialty care, basic DSME education, diabetes pamphlet	21.6%	Yes	HbA1c: 3×’s: baseline; 6 mos; 12 mos; QOL: N/A
Samuel-Hodge et al., 2009 [39]	201	100	64%	59	RCT	Church-based DSME: 1 individual counseling visit;12 bi-weekly group sessions; 12 monthly phone contacts; 3 encouragement postcards. Providers: peer counselor, dietician, other health care providers	Minimal care: direct mailings of 2 pamphlets, and 3 bimonthly newsletters to controls	13.7%	Yes	HbA1c: 3×’s: baseline, 8 mos, 12 mos; QOL: N/A
Skelly et al., 2005 [40]	41	100	100	62	RCT	4 bi-weekly home visits lasting approximately 1 h; 4 Diabetes Symptom-Focused Management Intervention modules. Provider: nurse	2 pre-intervention visits; 1 phone call; 1 final evaluation visit.	8.5%	Yes	HbA1c: 2×’s: baseline, within 1 month of treatment completion QOL: 2×’s: baseline, final evaluation; (Tools: Diabetes Symptom Distress Scale (DSDS), Quality of Life in Diabetes Instrument).
Walker et al., 2010 [41]	195	100	80.35%	60	QE	Three 2-h DSME sessions with inclusion of healthy snacks. Providers: diabetes educators	Usual care	Not specified	Yes	HbA1c: 2×’s: baseline, 5 months later QOL: 2 - 3×’s; (Tools: Problem Areas in Diabetes Survey -baseline, after completion of 3 sessions, 5 months later).

DSME interventions were heterogeneous in terms of setting, structure, content, contact hours, and provider type. Settings included primary care offices, hospitals, community health centers, diabetes education centers, churches, and patient homes. Seven provided individual DSME, [32, 33, 35, 36, 38, 40, 42] four provided group DSME, [29, 30, 37, 41] and three utilized both individual and group sessions [31, 34, 39]. The number of contact hours varied from 4 to 27; in two studies the contact hours were not specified [36, 39]. In half of the interventions, DSME was delivered by one type of health professional, most commonly a diabetes educator or nurse educator; other studies utilized combinations of diabetes educators, nurse case managers, registered dieticians, pharmacists, peer educators, and community health workers. Attrition was addressed in all but one study [41]; the mean attrition rate was 22.1%.

Twelve of the fourteen interventions were described by their authors as culturally-adapted for African-Americans [29–33, 35–41]. Seven of these authors provided further descriptions of their cultural adaptations, which consisted primarily of incorporation of African-American dietary preferences in nutrition education and/or use of race-concordant diabetes educators, peer educators, or community health workers.

All studies included in the systematic review measured change in HbA1c % as an outcome; 10 studies compared changes in HbA1c for intervention participants versus usual care [29, 31–39]. Of these 10 studies, five reported HbA1c changes favoring the intervention group [32–35, 39]. Eight studies reported pre and post HbA1c means and standard deviations for both the intervention and control groups and were therefore eligible for the HbA1c meta-analysis [29–32, 34, 36, 38, 39].

Eight studies included in the systematic review measured QOL as an outcome, with several using multiple QOL assessment tools. QOL measures included Mental Well Being and Social Well Being, [36] Psychological Distress Scale, [37] Healthy Days Measure Scale, [37] the 12-Item Short-Form Survey, (SF-12) [34] the Problem Areas in Diabetes Survey (PADS), [41, 42] the Diabetes Care Profile (DCP), [30] the Diabetes Empowerment Scale Short-Form (DESSF), [30] Diabetes Attitude Scale, [30] Diabetes Symptom Distress Scale, [40] and Quality of Life in Diabetes [40]. Five of these studies compared changes in QOL for intervention participants compared to usual care [30, 33, 39, 40, 42]. Four reported statistically significant improvements in intervention participants’ QOL compared to usual care, including improved physical, [33] mental, [33, 39, 40] and social well-being [40] and improved psychosocial adjustment [42]. Only two studies included pre- and post-intervention QOL means and standard deviations, and these studies used different QOL tools. Use of the standardized mean difference for comparing different patient-reported outcomes such as QOL score in a meta-analysis is cautioned against because the responsiveness of different QOL instruments to change may vary dramatically [43]. Therefore, QOL results were ultimately not combined in a meta-analysis.

## Meta-analysis results

**Fig. 2:**
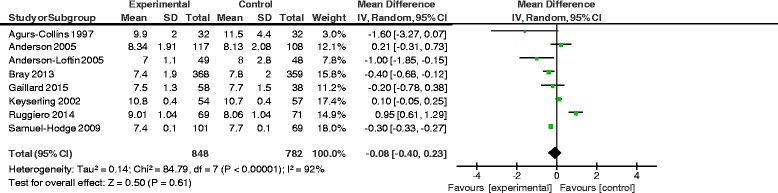
Forest Plot for HbA1c Meta-Analysis

Figure 2 shows the forest plot for HbA1c in DSME participants versus usual care. First, a random-effects meta-analysis was conducted to assess possible baseline HbA1c differences between intervention and control groups; a non-significant mean baseline HbA1c difference was observed: 0.1% [95% CI -0.25-0.5%]. A second random-effects meta-analysis estimated the WMD in HbA1c in the intervention versus usual care group post-intervention. The HbA1c WMD between intervention and usual care participants was not significant: 0.08% [− 0.40–0.23]; heterogeneity was high: *χ*^2^ = 84.79 (*p* < .001), *I*^2^ = 92% (*n* = 1630). Subgroup analyses of HbA1c by intervention versus usual care for culturally tailored interventions, individual versus group curriculum, intervention contact hours (< 10 versus ≥10), provider type, and attrition rate (< 20% versus ≥20%) were also non-significant (Table 2).

**Table 2:** HbA1c Meta-Analysis Subgroup Analyses

Variable	No. of Studies	Mean HbA1c Reduction	95% Confidence Interval	Cochran Q; *P* Value	*I* ^2^
Culturally tailored DSME					
Yes	7	−.07	[−.41,0.27]	84.73, <.001	93%
No	1	−.20	[−.78,.38]	N/A	N/A
DSME curriculum delivery					
Individual	3	.12	[−.81,1.06]	36.33, <.001	94%
Group	2	−.35	[−1.53,.08]	5.65, .02	82%
Combination	3	−.18	[−.57,.21]	28.98, <.001	93%
Intervention contact hours					
< 10	2	−.13	[−.62,.35]	9.36, .002	89%
≥ 10	6	−.15	[−.73,.44]	58.69, <.001	91%
DSME provider type					
Single-type	2	.02	[−1.89,1.92]	17.37, <.001	94%
Multiple	6	−.17	[−.42,.08]	33.12, <.001	85%
Attrition rate					
< 20%	5	−.17	[−.44,.11]	33.05, <.001	88%
≥ 20%	3	−.03	[−1.17,1.10]	24.03, <.001	92%

## Bias and quality assessment results

For most types of bias assessed, the risk of bias was low for the majority of studies included in the systematic review. Risk of selection bias due to random sequence generation was high for two studies and unclear for three; bias sue to allocation concealment was unclear for 10 studies. One study had unclear performance bias, all studies had low detection bias, one had unclear reporting bias, and none had other biases detected. The complete risk of bias ratings for included articles may be found in Additional file 2.

Given that the majority of included studies were randomized-controlled trials, the overall evidence was initially assessed as high-quality per GRADE criteria. One point was deducted from the evidence quality for heterogeneity of the study findings. As noted earlier, due to the small number of studies publication bias could not be assessed. Ultimately, the quality of the evidence was graded as moderate.

## Discussion

This meta-analysis found no significant impact of DSME on HbA1c in African-American DSME participants. This finding contrasts with prior DSME meta-analyses that have found HbA1c reductions ranging from 0.44–0.76% in the general population [14, 15] to 0.31% in DSME targeted at ethnic minorities [18]. The subgroup analysis of < 10 versus ≥10 contact hours also contrasts with a prior meta-analysis [14]; although Ricci-Cabello et al. found that effects did not vary by contact hours or intervention intensity [18]. The similarity of HbA1c outcomes for individual versus group DSME is consistent with prior meta-analyses [14, 18]. Likewise, the variation in DSME settings, delivery methods, intensity and contact hours is similar to the findings of other DSME meta-analyses [14, 18]. The high heterogeneity of HbA1c changes (*I*^2^ = 92%) may be a result of the substantial variations in these intervention characteristics.

The smaller number of DSME interventions measuring QOL relative to HbA1c is consistent with prior DSME meta-analyses, which have found a greater focus on surrogate outcomes such as HbA1c rather than patient-reported outcomes such as QOL [14]. It is promising that four of the five studies measuring QOL found statistically significant improvements in participants’ QOL versus controls; however, no studies explained if the statistically significant differences in QOL scores translated into clinically meaningful QOL improvements for patients.

The variety of QOL scales used likely reflects that QOL is a complex construct without a universal definition; however, this variety and the small number of DSME studies measuring QOL hampers the ability to compare findings across studies. In order to better understand the impact of DSME on QOL, more DSME studies should include QOL measures, which would allow for eventual pooling of studies using the same/similar QOL measures in meta-analyses. Future DSME research could also examine with relationship of QOL and potential moderators such as self-efficacy and social support [44].

Notably, in a subgroup analysis culturally-adapted DSME interventions did not yield better HbA1c results than non-culturally-adapted DSME. Prior DSME meta-analyses have not compared culturally to non-culturally tailored DSME. In Nam et al.’s meta-analysis of culturally-tailored DSME in ethnic minorities, the authors noted that more research was needed to determine the most effective culturally-tailored elements for various racial and ethnic groups [17]. Similarly, for a number of the studies in our meta-analysis it was difficult to ascertain the types of cultural adaptations made, and it was unclear whether certain features—such as use of race-concordant educators or recipe modifications—had a greater impact than others. More detailed guidelines are needed for the development and evaluation of culturally-adapted DSME in specific populations. Future research should also more rigorously assess approaches to cultural adaptations of DSME for African-Americans and the relative effectiveness of various culturally-tailored approaches. Furthermore, the included studies in this meta-analysis did not explicitly address social and systems-level contributors to diabetes disparities in African-Americans, such as socioeconomic status, racial discrimination or mistrust in the medical system. When developing DSME in the United States and globally, educators should be sensitive to the experiences of marginalized groups and how these experiences can impact diabetes self-management [45].

### Strengths and limitations

Our study has a number of strengths: it is the first systematic review and meta-analysis to examine the impact of DSME on HbA1c and QOL in African-American participants, and to include a subgroup analysis of the impact of culturally versus non-culturally tailored DSME on HbA1c in African-Americans. The study also benefitted from a prospectively-created study protocol utilizing a comprehensive search strategy that included hand searching of selected journals and grey literatures searches. Additionally, the protocol did not apply search restrictions based on publication year or language, which helped to ensure that all relevant interventions were captured. Of the fourteen studies in the systematic review, 10 were RCTs. strengthening the internal validity. For all risks of bias assessed, the majority of studies had low risk of bias, and the overall body of evidence was rated to be moderate quality per the GRADE criteria.

Limitations included the high risk of bias in random sequence generation for the two quasi-experimental studies included in the systematic review, and the unclear risks of bias across several studies, particularly for allocation concealment. Additionally, the HbA1c results had significant heterogeneity, as reflected by the large CIs and *I*^2^ value. Although subgroup analyses were performed, the small number of studies (*n* = 8) eligible for inclusion in the HbA1c meta-analysis limits the ability to draw conclusions about the optimal DSME intensity and delivery methods for African-Americans. The smaller number of articles measuring QOL(*n* = 5) and the inability to pool studies also warrants caution for drawing conclusions for the relationship between DSME and QOL in African-Americans. Finally, the limited number of studies included in the meta-analysis precluded assessment of publication bias. However, publication bias typically results in studies with significant findings being more likely to be published. Since the HbA1c meta-analysis was non-significant, this may lessen the possibility of publication bias in the included studies.

In addition, participants in the included studies may not be fully representative of the African-American population. For instance, in all of the studies the majority of participants were female; therefore, the findings may be less applicable to African-American men. Most studies reported limited socioeconomic status information, such as education level or income data; how these characteristics were measured varied across studies, making it difficult to compare these samples to the socioeconomic status of the African-American population. This may limit the external validity of the findings.

## Conclusion

Significant disparities remain in type 2 diabetes prevalence and outcomes among African-Americans, and DSME is recommended as part of standard type 2 diabetes care. Our study adds to the body of knowledge of the impact of DSME in African-Americans by showing a non-significant impact on HbA1c in African-American participants. The high levels of heterogeneity in the HbA1c findings--as evidenced by wide CIs and *I*^2^ values—demonstrate a need for more rigorously-designed DSME trials for African-Americans and further research to understand what DSME intervention characteristics, if any, consistently contribute to improved HbA1c in this population. Finally, the smaller number of interventions measuring QOL indicates the need for greater prioritization of QOL and other patient-important outcomes in future DSME research among African-Americans.

## Additional files

Additional file 1: OVID Medline search strategy. (DOC 29 kb)

Additional file 2: Risk of bias ratings for included studies. (DOC 46 kb)

## Abbreviations

AADE: American Association of Diabetes Educators
ADA: American Diabetes Association
CI: confidence interval
DAWN2: Diabetes Attitudes, Wishes, and Needs second study DSME diabetes self-management education
DQOL: diabetes quality of life
GRADE: Grading of Recommendations, Assessment, Development, and Evaluation criteria
HbA1c: hemoglobin A1c
HRQL: health-related quality of life
MeSH: medical subject headings
NIDDM: non insulin-dependent diabetes mellitus
PICO: patient, intervention, comparison, outcome PRISMA Preferred Reporting Items for Systematic Reviews and Meta-Analyses
PROSPERO: international prospective register of systematic reviews
QOL: quality of life
SF-36: short-form 36
T2DM: type 2 diabetes mellitus
WHOQOL: World Health Organization Quality of Life
WMD: weighted mean difference
