# Supplementary material for: The effect of diabetes self-management education on HbA1c and quality of life in African-Americans: a systematic review and meta-analysis
Source: BMC Health Serv Res. 2018 May 16;18:367. doi: 10.1186/s12913-018-3186-7 (PMC5956958; doi:10.1186/s12913-018-3186-7)
Supplement: Supplementary file 1 — OVID Medline search strategy. (DOC 29 kb) [file 12913_2018_3186_MOESM1_ESM.doc]

Additional file 1 Ovid Medline Search Strategy (no language or date limits)

| 1. African Americans.af. or "African Americans".mh. |  |
| --- | --- |
| 2. (type 2 diabetes or type 2 diabetes mellitus or diabetes or T2DM).af. or "Diabetes Mellitus".sh. or "Diabetes Mellitus, Type 2".sh. or NIDDM.af. or Non-insulin dependent diabetes mellitus.af. |  |
| 3. 1 and 2 |  |
| 4. (diabetes self management education or self management education or DSME or health education or diabetes education).af. or "Patient Education as Topic".sh. or "Self Care".sh. |  |
| 5. 3 and 4 |  |
| 6. (randomized controlled trial or controlled clinical trial).pt. or randomly.af. or randomized.af. or control.af. or trial.af. or groups.af. or quasi-experimental.af. |  |
| 7. 5 and 6 |  |
| 8. (HbA1c or A1c or "glycemic control").af. or "Hemoglobin A1c, glycosylated".sh. or "Hemoglobin A1c protein, human".sh. or HRQOL.af. or QoL.af. or health-related quality of life.af. or "Quality of Life".sh. or QoL tools.af. or questionnaires.af. or surveys.af. or SF-36.af. or WHOQOL.af. or DQOL.af. or well-being.af. or psychological well-being.af. or emotional well-being.af. |  |
| 9. 7 and 8 |  |
