# Supplementary material for: The effect of diabetes self-management education on HbA1c and quality of life in African-Americans: a systematic review and meta-analysis
Source: BMC Health Serv Res. 2018 May 16;18:367. doi: 10.1186/s12913-018-3186-7 (PMC5956958; doi:10.1186/s12913-018-3186-7)
Supplement: Supplementary file 2 — Risk of bias ratings for included studies. (DOC 46 kb) [file 12913_2018_3186_MOESM2_ESM.doc]

Additional file 2 Risk of Bias Ratings

| Study | Random sequence  generation  (selection bias) | Allocation  concealment  (selection bias) | Blinding of participants  and personnel  (performance bias) | Blinding of outcome  assessment  (detection bias) | Incomplete outcome  data  (attrition bias) | Selective reporting  (reporting bias) | Other bias |
| --- | --- | --- | --- | --- | --- | --- | --- |
| Agurs-Collins TD, Kumanyika SK, Ten Have TR, Adams-Campbell LL. A randomized controlled trial of weight reduction and exercise for diabetes management in older African-American subjects. Diabetes Care 1997 Oct;20(10):1503-1511. | Unclear | Unclear | Low | Low | Unclear | Low | Low |
| Amoako E, Skelly AH, Rossen EK. Outcomes of an intervention to reduce uncertainty among African American women with diabetes. West J Nurs Res 2008;30(8):928-942. | Low | Low | Low | Low | Low | Low | Low |
| Anderson, R. M., Funnell, M. M., Nwankwo, R., Gillard, M. L., Oh, M., & Fitzgerald, J. T.. Evaluating a problem-based empowerment program for african americans with diabetes: Results of a randomized controlled trial. Ethnicity & Disease 2005;15(4):671-678. | Unclear | Unclear | Low | Low | Unclear | Low | Low |
| Anderson-Loftin, W., Barnett, S., Bunn, P., Sullivan, P., Hussey, J., & Tavakoli, A.. Soul food light: Culturally competent diabetes education. Diabetes Educator 2005;31(4):555-563. | Low | Low | Low | Low | Low | Low | Low |
| Bray P, Cummings DM, Morrissey S, Thompson D, Holbert D, Wilson K, et al. Improved outcomes in diabetes care for rural African Americans. Ann Fam Med 2013 Mar-Apr;11(2):145-150. | Low | Unclear | Low | Low | Low | Low | Low |
| Carter EL, Nunlee-Bland G, Callender C. A patient-centric, provider-assisted diabetes telehealth self-management intervention for urban minorities. Perspectives in health information management 2011;8:1b. | Low | Unclear | Low | Low | Unclear | Low | Low |
| Gaillard T, Amponsah G, Osei K. Patient-Centered Community Diabetes Education Program Improves Glycemic Control in African-American Patients with Poorly Controlled Type 2 Diabetes: Importance of Point of Care Metabolic Measurements. Journal of National Black Nurses Association 2015 Jul;26(1):50-57. | Unclear | Unclear | Low | Low | Unclear | Unclear | Low |
| Gary TL, Batts-Turner M, Bone LR, Yeh HC, Wang NY, Hill-Briggs F, et al. A randomized controlled trial of the effects of nurse case manager and community health worker team interventions in urban African-Americans with type 2 diabetes. Control Clin Trials 2004;25(1):53-66 | Low | Unclear | Low | Low | Low | Low | Low |
| Keyserling, T. C., Samuel-Hodge, C. D., Ammerman, A. S., Ainsworth, B. E., Henríquez-Roldán, C. F., Elasy, T. A., ... & Bangdiwala, S. I.. A randomized trial of an intervention to improve self-care behaviors of African-American women with type 2 diabetes.. Diabetes care 2002;25(9):1576-1583. | Low | Low | Low | Low | Low | Low | Low |
| Peña-Purcell NC, Jiang L, Ory MG, Hollingsworth R. Translating an evidence-based diabetes education approach into rural African- American communities: The "wisdom, power, control" program. Diabetes Spectrum 2015;28(2):106-115. | High | Unclear | Unclear | Low | Unclear | Low | Low |
| Ruggiero L, Riley BB, Hernandez R, Quinn LT, Gerber BS, Castillo A, et al. Medical assistant coaching to support diabetes self-care among low-income racial/ethnic minority populations: Randomized controlled trial. West J Nurs Res 2014;36(9):1052-1073. | Low | Unclear | Low | Low | Low | Low | Low |
| Samuel-Hodge CD, Keyserling TC, Park S, Johnston LF, Gizlice Z, Bangdiwala SI. A randomized trial of a church-based diabetes self-management program for African Americans with type 2 diabetes. Diabetes Educ 2009 May-Jun;35(3):439-454. | Low | Low | Low | Low | Low | Low | Low |
| Skelly AH, Carlson JR, Leeman J, Holditch-Davis D, Soward ACM. Symptom-focused management for African American women with type 2 diabetes: a pilot study. Appl Nurs Res 2005 11;18(4):213-220. | Low | Unclear | Low | Low | Unclear | Low | Low |
| Walker EA, Stevens KA, Persaud S. Promoting diabetes self-management among African Americans: an educational intervention. J HEALTH CARE POOR UNDERSERV 2010 08/02;21(3):169-186. | High | Unclear | Low | Low | Unclear | Low | Low |
